# Robustness and Stability of the Gene Regulatory Network Involved in DV Boundary Formation in the *Drosophila* Wing

**DOI:** 10.1371/journal.pone.0000602

**Published:** 2007-07-11

**Authors:** Javier Buceta, Héctor Herranz, Oriol Canela-Xandri, Ramon Reigada, Francesc Sagués, Marco Milán

**Affiliations:** 1 Centre especial de Recerca en Química Teòrica (CeRQT), Parc Científic de Barcelona, Barcelona, Spain; 2 Institució Catalana de Recerca i Estudis Avançats (ICREA) and Institute for Research in Biomedicine (IRB), Parc Científic de Barcelona, Barcelona, Spain; 3 Departament de Química-Física, Universitat de Barcelona, Barcelona, Spain; Center for Genomic Regulation, Spain

## Abstract

Gene regulatory networks have been conserved during evolution. The *Drosophila* wing and the vertebrate hindbrain share the gene network involved in the establishment of the boundary between dorsal and ventral compartments in the wing and adjacent rhombomeres in the hindbrain. A positive feedback-loop between boundary and non-boundary cells and mediated by the activities of Notch and Wingless/Wnt-1 leads to the establishment of a Notch dependent organizer at the boundary. By means of a Systems Biology approach that combines mathematical modeling and both *in silico* and *in vivo* experiments in the *Drosophila* wing primordium, we modeled and tested this regulatory network and present evidence that a novel property, namely refractoriness to the Wingless signaling molecule, is required in boundary cells for the formation of a stable dorsal-ventral boundary. This new property has been validated *in vivo*, promotes mutually exclusive domains of Notch and Wingless activities and confers stability to the dorsal-ventral boundary. A robustness analysis of the regulatory network complements our results and ensures its biological plausibility.

## Introduction

As occurs in most biological phenomena, gene expression underlies morphogenesis. By means of gene expression, cells specialize for shaping and organizing tissues. This feature poses the interesting question of how cell fate is determined, i.e. how a given cell and its progeny “know” what genes should and should not be expressed in order to perform a particular task. The latter immediately suggests the concept of information and reveals an additional function performed by gene expression and regulated by cell interactions: gene expression provides positional information [1]. Thus, genetic activity establishes an expression pattern, a “map”, by means of which cell fate is determined depending on the relative positions of cells inside the tissue. This orchestrated plan sets up a dynamical coordinate system that links the gene expression pattern, genotype, to the resulting biological structure, phenotype.

**Figure 1:**
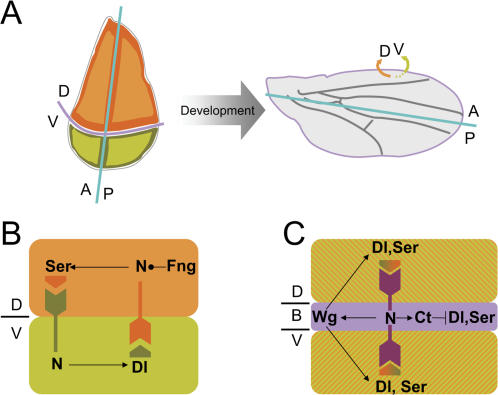
Gene regulatory network involved in DV boundary formation. (A) The wing primordium (left) is subdivided into anterior (A) and posterior (P) compartments, as well as into dorsal (D) and ventral (V) compartments, which will give rise to specific biological structures within the adult wing (right). The compartments are named after the position that their cells and progeny will occupy by the end of development. (B, C) Early in development (B), Serrate (Ser) signals to V cells to activate Notch (N). Likewise, Delta (Dl) signals to D cells to activate Notch modified by Fringe (Fng) along the DV boundary. Later in development (C), ligands expression becomes symmetric with respect to the boundary and a positive feedback-loop between Wingless (Wg) and Ser/Dl-expressing cells maintains the signaling center along the DV boundary. Notch activity elicits Cut expression that represses Dl and Ser in boundary cells.

The wing primordium of *Drosophila* and the rhombomeres of the vertebrate hindbrain provide well-characterized examples in which domains of gene expression, modulated by short and long-range cell interactions, link to well-defined biological structures. Both systems become subdivided into stable cell populations called compartments, which do not mix during development ([2], [3], Figure 1A). Compartment subdivision is induced primarily by the specific expression and activity of transcription factors that confer a compartment specific fate (reviewed in [4]). Short-range cell interactions between adjacent compartments lead to the expression of long-range signaling molecules at the compartment boundaries, thus serving these boundaries as signaling centers with long-range organizing properties.

The *Drosophila* wing primordium and the rhombomeres of the vertebrate hindbrain also share the gene network that establishes and maintains the stability of the compartment boundary. Activation of the receptor Notch at this boundary, due to the activity of the Notch ligands in nearby cells, induces the expression of the signaling molecules Wingless (Wg) and Wnt-1 in boundary cells of the fly wing and the vertebrate hindbrain, respectively ([5]–[8], Figures 1B and 1C). Wg or Wnt-1 maintain the expression of Notch ligands, thus establishing a positive feedback loop and ensuring high activity of Notch at the compartment boundaries [8]–[10]. Notch activity then regulates growth of the surrounding non-boundary cells and is required for maintaining the lineage restriction boundary [11]–[14].

A distinctive feature of the process that leads to stable localization of the Notch-dependent organizer at the dorsal-ventral (DV) compartment boundary is the refinement of the Notch activation domain to a thin stripe with a final width of two-three cells. This process is mediated by the activity of Wg [15] and it is carried out in two different ways. In the first, high levels of Wg signaling induce the expression of Notch ligands Serrate and Delta which repress Notch signaling in a cell-autonomous manner [9], [10]. Co-expressed Serrate and Delta interact with Notch and form heteromeric complexes that are not found at the cell surface [16]. The activity of Notch at the boundary induces expression of the homeobox gene *cut* in boundary cells [10] which represses expression of *Delta* and *Serrate* [9]. Thus, boundary cells are alleviated from Serrate and Delta dependent Notch repression. In the second, Dishevelled, a cytoplasmic mediator of the Wg signaling pathway, binds the intracellular domain of Notch and, as a consequence, interacts antagonistically with it, blocks Notch signaling, and reduces the receptor activity [17]. How boundary cells become refractory to the negative activity of Dishevelled remains to be addressed so far.

Parallel to the experimental efforts made to elucidate gene regulatory interactions, mathematical modeling approaches have become an increasingly powerful tool due to their predictive and analytic capabilities [18]. Recent successes in modeling include the prediction of phenotypes [19], the functioning of the Epidermal-Growth-Factor receptors [20], the determination of the left-right axis in vertebrates [21], [22] and the formation of robust gradients [23], [24]. In the context of DV boundary formation of the *Drosophila* wing, continuous [25] and, more recently, Boolean [26] regulatory networks have also been proposed. Unfortunately, these models did not consider all the aforementioned properties of the system, like the repression of Notch by the activity of Wg or the diffusion of Wg in the case of a Boolean description.

Here we revise the gene regulatory network for the establishment and maintenance of the DV boundary in the *Drosophila* wing. We take a Systems Biology approach and benefit from the feedback between our *in silico* and *in vivo* experiments to model and test the network interactions. Most importantly, our modeling approach takes into account all the properties of the system described so far, including intra- and inter-cellular Notch-ligand binding events, Wg morphogen diffusion, and regulatory interactions between species in a spatially extended system that comprises a large number of cells mimicking the wing primordium. As a main novelty, we present *in silico* evidence that a new property is required in boundary cells for stable maintenance of the organizing centre: namely, boundary cells must be refractory to the Wg signal. This refractoriness has been experimentally validated in the wing primordium, mediates the regulatory interplay between Notch and Wg and promotes the formation of mutually exclusive domains in terms of their activities. Consequently, it becomes responsible for size regulation of the boundary cell population and for the polarized signaling of the ligands towards the boundary. We present *in vivo* evidence that this property is defined by the activity of Notch through its target gene *cut*. Thus, Cut activity makes boundary cells not only refractory to Serrate and Delta expression [9] but also to any negative input coming from Wg, thus allowing stable Notch activation [10].

Within our modeling approach, we have also introduced novel *in silico* experiments such as mosaic analysis, where the behavior of mutant and neighboring cells can be analyzed. Comparison between these *in silico* results and their *in vivo* counterparts allowed us to check whether the gene interactions were appropriately defined and weighted, thereby ensuring an accurate design of the regulatory network “circuitry”. In addition, the analysis of the dynamics of DV boundary formation in various genetic backgrounds has been also addressed. In this regard we have been able to predict the dynamical behavior of relevant mutant genotypes and also to shed light on a distinctive dynamical property of boundary formation, namely the Notch activity refinement. Finally, we have introduced a robustness analysis that shows that the proposed regulatory network is remarkably stable with respect to stochastic, yet biologically plausible, parameter variation and noisy perturbations of production rates.

## Results and Discussion

### Modularity: spatio-temporal frame of the developmental process of interest

Modularity is crucial for understanding the emergence of biological complexity [27]. Functional modules constitute the building blocks of a developing organism and their proper connections give rise to an adult organism. The establishment of the DV organizer in the *Drosophila* wing provides a beautiful example of a functional module where several processes sequentially act to shape the boundary. Thus, in order to appropriately frame-in the functional module that drives the developmental process of our interest, we review the stages that lead to the establishment of the DV boundary in the *Drosophila* wing (Figure 1).

Expression of the LIM-homeodomain transcription factor Apterous in D cells of the early wing primordium activates the expression of the Notch ligand Serrate and the glycosyltransferase Fringe and restricts expression of Delta, another Notch ligand, to V cells [5], [7], [28]–[30]. Fringe modifies the receptor Notch and makes D cells more sensitive to Delta and less sensitive to Serrate [31], [32]. Unmodified Notch in V cells responds better to Serrate than to Delta (Figure 1B). The preferential response of the receptor Notch to the ligand expressed in the opposite compartment ensures activation of the Notch pathway only at the DV boundary. Notch activation induces Serrate and Delta expression, thus leading to a positive feedback loop that transiently maintains each others expression (Figure 1B, [32]). Later, an increase in dLMO levels, a LIM domain-containing protein that competes with Apterous to bind its cofactor Chip, leads to a reduction in Apterous protein activity [30], [33]–[35]. Accordingly, Apterous dependent expression of Fringe gets lost. The reduction in Apterous and Fringe activities has two other main consequences: the compartment-specific expression of Delta and Serrate ligands ceases and Notch can respond equally to both ligands. The initial condition of the developmental process we aim to model corresponds to this point in time.

The positive feedback loop between Notch and its ligands leads to increased levels of Notch activity at the boundary. Above a particular threshold, activity of the Notch receptor causes the expression of the signaling molecule Wg first and Cut later ([5], [7], [10], Figure 1C). Wg induces Serrate and Delta expression in nearby non-boundary cells, which can signal back to Notch [9], [10]. This positive feedback loop between boundary and non-boundary cells ensures activation of Notch and expression of Wg at the DV boundary. Additionally, Cut represses the expression of Delta and Serrate (Figure 1C) helping to maintain Notch activity and Wg expression in boundary cells [9], [10]. This stage constitutes the endpoint of the developmental dynamics of our interest.

### *In silico* modeling of DV boundary formation

**Figure 2:**
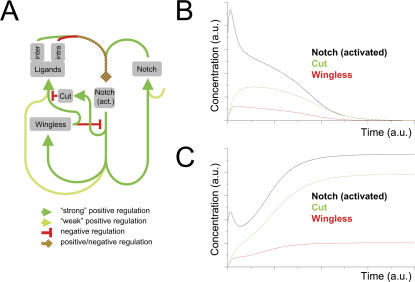
*In silico* testing of the gene regulatory network: refractoriness to Wg in boundary cells renders stability to the DV regulatory network. (A) Regulatory network for the formation of the DV boundary. Positive and negative regulations are coded with green and red colors, respectively. Color intensity in positive regulations indicates, qualitatively, the strength of expression levels (the lighter the weaker). The green-red dashed line that ends with a rhombic arrowhead indicates that receptor-ligand dynamics may lead to either positive or negative regulation: Notch-ligand binding in the same cell (intra) or in adjacent neighboring cells (inter) lead, respectively, to titration (sequestering effects) or activation of Notch. Activated Notch induces ligand and receptor expression at low levels, thus closing a positive feedback loop that maintains each other's expression at early stages of wing development. Note that Notch has an additional autonomous off-network regulation. Increased Notch activity induces expression of Wg and Cut. The latter represses Ser and Dl. (B) Evolution of Wg (red) expression levels, Notch activated (black), and Cut (green) in boundary cells as a function of time. The boundary is initially established but cannot be maintained (see text). (C) Evolution of Wg (red) expression, Notch activated (black), and Cut (green) levels in boundary cells as a function of time in a scenario in which refractoriness to Wg has been taken into account in boundary cells (see text). Note stable activation of Notch and expression of Wg and Cut in this case, when compared to (B).

On the basis of the aforementioned regulatory interactions, we aimed to revise, model and test the network that drives the establishment and maintenance of the DV organizer (Figure 2A). Our approach took into account a reduced, yet realistic, set of elements and their regulatory relationships. The main features of the network are the following: activated Notch is the “conductor” for the establishment of the DV boundary. Notch can be signaled by the ligands Serrate or Delta. Given that at this stage of development there is no difference in the way the ligands signal to Notch, we did not consider any difference between ligands apart from their initial DV asymmetric expression (Serrate:dorsal and Delta:ventral). Nonetheless, in our modeling approach we distinguished between the two ligands to track how symmetric expression is obtained at flanking stripes of the boundary. Depending on the relative concentration of receptor and ligands in the same cell (intracellular interactions) and neighboring cells (intercellular interactions), ligands may lead or not to the activation of the receptor. In Figure 2A we stress the dichotomous role played by receptor-ligand interactions (either positive or negative regulation of Notch pathway). If the Notch receptor is activated, then the transcription-translation of its downstream genes starts. Downstream genes are expressed, or not, at appreciable levels depending on the degree of Notch activity. As activation increases, Notch and the ligands themselves are expressed, afterwards Wg and then Cut, as experimentally shown elsewhere [10], [32], [36]. This ordered sequence of expression as a function of Notch activity levels fixes an ordered sequence for the threshold values of the regulatory functions in our modeling approach. Independently of Notch activation, there is an autonomous off-network Notch transcription-translation dynamics that keeps the expression levels of the receptor to a basal level in wing cells. Once Wg is expressed, it exerts its aforementioned roles in the proposed regulatory network: induction of Notch ligand expression and down-regulation of the Notch pathway. On the other hand, Cut represses Delta and Serrate expression in boundary cells.

We implemented this regulatory network in *in silico* cells. Our *in silico* wing primordium consisted of a hexagonal lattice of 1500 cells. Further details on the implementation of the model are provided in the Material and Methods section and in Protocols S1 and S2 of the Supporting Information. *In silico* experiments helped us to explore whether such a regulatory scheme succeeds in converting the initial low activity of Notch into the final peak of Notch activation and in generating a stable Wg morphogen gradient. Note that the *in silico* figures (and movies) show Senseless protein expression, although this species was not considered in our modeling approach. However, Senseless is a Wg activity reporter in the wing primordium [37] that can definitively be traced in our *in silico* experiments (see Supporting Information). Thus, we intentionally misused the name “Senseless” to label Wg activity for better comparison with the *in vivo* results (see below). Figure 2B shows a prototypical evolution of Notch, Wg and Cut expression levels in boundary cells as a function of time in *in silico* experiments. The initial dynamics is first driven by Notch-ligand mediated short-range cell interactions. At these early stages, an increase in Notch activity and Wg and Cut expression levels takes place. However, at later stages these die out. *In silico* experiments reveal this is the common scenario for the vast majority of parameter values. Nonetheless, we note that highly constrictive parameter tweaking allows for concomitant activities of Wg and Notch pathways in boundary cells. However, as shown below, this alternative is not observed in wild-type *in vivo* experiments. Moreover, parameter tweaking is in opposition to the underlying idea of a conserved, and supposedly robust, regulatory network. All together, these results suggest that either some elements or interactions are missing in the approach to DV boundary formation shown in Figure 2A since it fails to provide stable Notch activity at boundary cells.

### A new property is required for stable DV boundary formation

**Figure 3:**
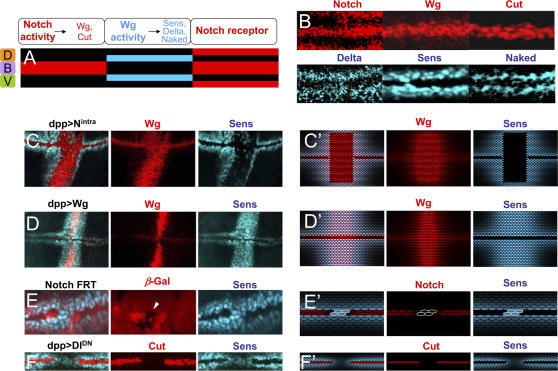
Refractoriness to Wg is defined by Notch. (A) Schematic representation of the results pointed out by (B) in regard of the expression pattern in DV boundary and neighboring cells. Several cell populations can be distinguished at and around the DV boundary in terms of the expression of Notch protein and the activity of Notch and Wg pathways. Orange, green, and violet squares highlight the D and V compartments, and the DV boundary, respectively. (B) High magnification of the DV boundary of mature wild-type wing discs showing, in red, Wg and Cut protein expression in boundary cells as well as higher levels of Notch protein expression in boundary and neighboring cells. Non-boundary cells show, in blue, high levels of Delta and Senseless (Sens) protein and *naked* mRNA. As revealed by Cut and Sens expression, the width of the boundary and flanking cell populations comprise a small number (two-three) of cells. (C,D) Mature wing discs expressing the intracellular domain of Notch, N^intra^, (C) or Wg (D) under *dpp^Gal4^* control. In the first case, Gal4-expressing cells do not respond to Wg whereas in the second case they do. (E) Small clones of cells in the boundary lacking *Notch* activity marked by the absence of GFP (red) and pointed out by means of a white arrowhead. Sens protein (in blue) starts to be expressed in boundary mutant cells. (F) Mature wing disc that expresses a dominant negative form of Delta (Delta^DN^) under *dpp^Gal4^* control. Note that boundary cells lacking Cut expression (in red) start to express Sens (blue). (C'–F') *In silico* counterparts of the results shown in (C–F). Ectopic expression of activation of Notch (C') or Wg (D'). (E'–F') As occurs *in vivo*, the absence of Notch activity in the boundary causes those cells to lose Cut expression and to start responding to Wg.

In the description and modeling of regulatory networks that drive developmental pattern formation, an over-simplified approach in terms of the genes involved and their interactions causes a misleading “circuitry”. In the context of DV boundary formation, the following observation suggests that a new property might have to be implemented to provide stability to the regulatory network: since Wg refines the width of the Notch activation stripe through inhibition of the Notch receptor by Dishevelled, stable activation of Notch at the boundary might require Wg to not negatively act on boundary cells. Indeed, Notch ligands Serrate and Delta, as well as other genes transcriptionally regulated by the activity of Wg (e.g. *senseless*, [37], see also *nemo, notum* and *naked*,[38]–[41]), are not expressed in boundary cells (Figures 3A and 3B), suggesting that the Wg signaling pathway is blocked in these cells.

We then tested whether after the implementation of this new property, refractoriness in boundary cells to Wg activity, our modeling approach succeeds in reproducing the late stationary pattern of gene expression and activity observed *in vivo*. To this end, we inhibited Wg activity in a two-cell wide stripe at the boundary. As shown in Figure 2C, in this case the initial dynamics driven by Notch-ligand short-range cell interactions evolves until it reaches a steady stage where Notch activation, Wg diffusive signaling, and Cut expression are self-sustained due to the interactions between boundary and non-boundary cells. DV boundary establishment and maintenance is pointed out by robust expression of active Notch, and also by the formation of a Wg morphogen gradient towards both compartments of the disc (data not shown). These results are in agreement with the observed *in vivo* pattern.

### Cut induces refractoriness to Wg in boundary cells

We have presented *in silico* evidence that refractoriness to the Wg signal in boundary cells provides stability to the gene regulatory network. Boundary cells are characterized by high levels of Notch activity, thus suggesting Notch is responsible for making boundary cells refractory to the Wg signal. We therefore analyzed *in vivo* the role of Notch in this process in the developing wing primordium. Ectopic activation of Notch in non-boundary cells represses Wg target gene expression (Figure 3C, see also Figure S1). Note that Notch, in this case, causes ectopic Wg expression in non-boundary cells, which induces target gene expression only in Wg non-expressing cells. By contrast, ectopic expression of Wg alone induces the expression of target genes in both Wg-expressing and non-expressing cells (Figure 3D and Figure S1). When boundary cells lack Notch activity, either by mutation or by expression of a dominant negative form of Delta known to titrate out the Notch receptor, these cells start to express target genes of Wg (Figures 3E and 3F, and also Figure S1). We can then conclude that either Notch activity itself, or one or several of its target genes inhibits the expression of Wg target genes in boundary cells.

**Figure 4:**
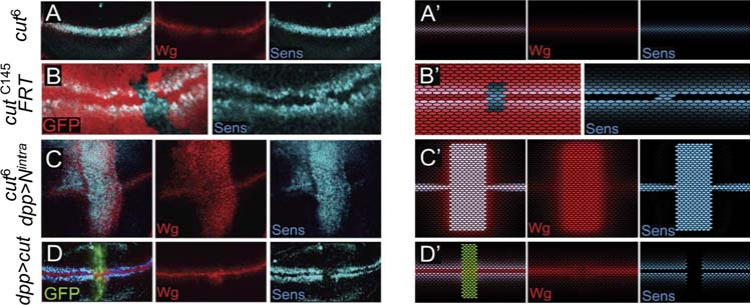
Refractoriness to Wg is conferred by the Notch target gene *cut.* (A) Mature *cut^6^* mutant wing disc showing co-expression of the proteins Senseless, Sens, (in blue) and Wg (in red) in boundary cells. (B) Clones of cells lacking *cut* activity marked by the absence of GFP (red). Sens (in blue) starts to be expressed in boundary cells. (C) Mature *cut^6^* mutant wing disc that expresses the intracellular domain of Notch (N^intra^) under *dpp^Gal4^* control. Wg protein expression is shown in red and Sens protein expression in blue. (D) Mature wing disc that expresses Cut and GFP (green) under *dpp^Gal4^* control. Note the loss of Sens expression (blue) and ectopic expression of Wg (red) in non-boundary cells. (A'–D') *In silico* counterparts of the results shown in (A–D).

High levels of Notch activity induce expression of the homeobox gene *cut* in boundary cells [10] and Cut has been previously shown to be required to repress *Delta* and *Serrate* expression in these cells [9]. We then examined whether Cut mediates the activity of Notch in inhibiting the expression of other Wg target genes. In the absence of Cut activity, either in a homozygous mutant background or in clones of mutant cells, boundary cells start expressing genes regulated by the Wg signal (Figures 4A and 4B, and Figure S1), and ectopic Notch activation in non-boundary cells is now unable to repress Wg target gene expression (Figure 4C; compare with Figure 3C; see also Figure S1). Note that Notch, in this case, causes ectopic expression of Wg, which induces target gene expression in both Wg-expressing and non-expressing cells. Finally, forced expression of Cut in non-boundary cells represses the expression of Wg target genes (Figure 4D and Figure S1). Taken together, these results indicate that Cut is not only required but also sufficient to inhibit Wg target gene expression in boundary cells downstream of Notch.

### Cut blocks the Wg pathway at the level or upstream of Armadillo

**Figure 5:**
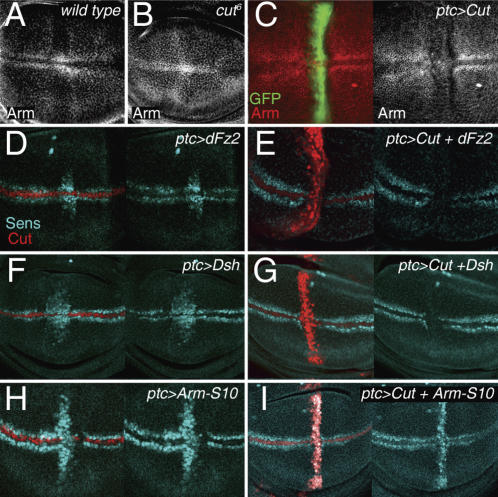
Cut blocks the Wg pathway at the level or upstream of Armadillo. (A–C) Mature *wild-type* (A), *cut^6^* (B) and *dpp^Gal4^*; *UAS-Cut UAS-GFP* (C) wing discs labeled to visualize Armadillo (Arm) protein expression levels (in white or red) and GFP (in green). (D–I) Mature wing discs that expressed dFz2 (D), Dsh (F), Arm^S10^ (H), dFz2 and Cut (E), Dsh and Cut (G), or Arm^S10^ and Cut (I) under the control of the *ptc^Gal4^* driver and labeled to visualize Senseless (Sens) protein expression levels (in blue) and Cut (in red).

Cut might exert its function either by blocking the Wg signaling pathway or, alternatively, by inhibiting the expression of every Wg target gene. The Wg signaling pathway is activated by controlling the levels and subcellular localization of the transcriptional co-activator Armadillo (Arm, known as ß-catenin in vertebrates; reviewed in [42]). In the absence of Wg signal, Arm levels are kept low through degradation. This degradation depends on the phosphorylation of Arm by the kinase Shaggy/Zeste white-3/Glycogen synthase kinase-3ß (GSK-3ß). Phosphorylated Arm is recognized rapidly by the proteasome and destroyed. Following Wg ligand binding, this degradation is inhibited, which enables Arm to accumulate, enter the nucleus and activate a transcriptional response. In the *Drosophila* wing, Arm protein levels are severely reduced in boundary cells, when compared with adjacent cells (Figure 5A), even though extracellular Wg protein is available in both types of cells. This observation indicates that the activity of the Wg signaling pathway is repressed in these cells at the level or upstream of Arm. Consistent with this observation, a dominantly activated form of Arm (Arm^S10^), which lacks the GSK-3ß phosphorylation sites and escapes degradation, induces expression of Wg targets in boundary cells (Figure 5H). Overexpression of any other limiting factor of the Wg pathway that acts upstream of Arm is unable to induce Wg target gene expression in these cells (Figures 5D and 5F).

Cut appears to mediate this type of repression of the Wg signaling pathway. In the absence of Cut activity, Arm protein levels are not reduced in boundary cells (Figure 5B), and ectopic expression of Cut in non-boundary cells reduces Arm protein levels and represses the expression of Wg target genes (Figure 5C and 4D, respectively). Moreover, Arm^S10^ can bypass the effects of ectopic Cut expression and restores Wg target gene expression in non-boundary cells (Figure 5I). Co-expression of limiting factors of the Wg pathway acting upstream of Arm does not cause this effect (Figures 5E and 5G). Taken together, these results indicate that Cut blocks the Wg signaling pathway at the level or upstream of Arm. Cut might exert its function through transcriptional regulation of a gene product involved in regulating the degradation of Arm.

### Cut renders stability to the DV boundary

**Figure 6:**
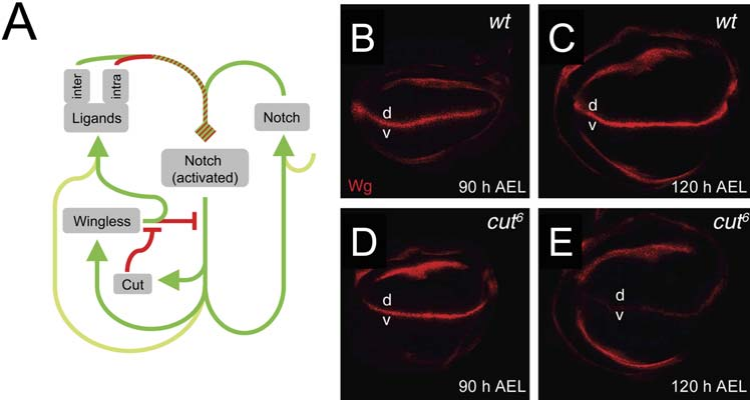
Proposed regulatory network for the establishment of the DV boundary: Cut renders stability to the DV boundary. (A) Proposed regulatory network for stable formation and maintenance of the DV boundary. The network includes the capacity of boundary cells, defined by the activity of Cut, to block the Wg signaling pathway at all levels (both positive and negative regulations). (B–E) Wg expression in *wild-type* (B, C) or *cut^6^* mutant (D, E) wing discs at 90 hr (B, D) or at 120 hr (C, E) after egg laying (AEL).

So far we have provided *in vivo* evidence that Cut is required in boundary cells to repress the Wg signaling pathway and also, by means of *in silico* experiments, that such repression leads to a stable DV boundary formation. Figure 6A summarizes both results and shows our proposal of regulatory network for the establishment of the DV boundary. As expected, *in silico* implementation of the refractoriness to the Wg signal via Cut leads to stable DV boundary formation and reproduces the results shown in Figures 3B. The stationary pattern of gene expression and activity observed in this case is in agreement with *in vivo* results (see Figure S3; see also Movie S2).

We can also extend further our conclusions with regards to the role played by Cut in DV boundary formation. As mentioned before, in the absence of refractoriness to the Wg signal (provided by the activity of Cut in boundary cells) an initial increase in Notch activity and Wg expression takes place (see Figure 2). This result suggests that Cut is dispensable for the onset of the DV boundary. This and the evolution predicted by our modeling (see Movie S1) are in agreement with the *in vivo* results (Figures 6B-6E, see also [10]). In *cut^6^* mutant discs, the early activation of Notch at the DV boundary, as shown by the expression of Wg, is comparable to wild-type discs (compare Figures 6B and 6D). However, in mature third instar discs Notch activity and Wg expression are not maintained in the mutant background (compare Figures 6C and 6E). Taken together, these results indicate that refractoriness of boundary cells to the Wg signal provided by the activity of Cut is required to shape a stationary and stable DV boundary in the developing wing primordium.

### *In silico* testing of the regulatory network

We further tested the proposed regulatory network by *in silico* experiments. These consisted either in inducing the ectopic expression or activation of a particular signaling molecule or pathway or in removing their activities in the whole wing primordium or in a subset of cells, and monitoring the behavior of these and surrounding cells. We compare the results obtained with their *in vivo* counterparts. In addition, these experiments also allowed us to fine-tune and estimate the value of some parameters in the proposed network. Results are shown in Figures 3C'-3F' and 4A'-4D'.

In the first type of experiments, expression of Wg and Senseless was monitored after ectopic activation of the Notch signaling pathway (Figure 3C') or ectopic expression of Wg (Figure 3D') in a stripe perpendicular to the DV boundary. High levels of Notch activity induce Wg expression but simultaneously cause cells to be refractory to the Wg signal. Thus, Senseless is expressed only in Wg non-expressing cells, which show no Notch activity (Figure 3C'). In contrast, ectopic expression of Wg alone induces Senseless expression in Wg-expressing and non-expressing cells where there is a lack of Notch activity (Figure 3D').

In the second type of experiments, in our *in silico* lattice of cells, we generated a subset of cells that lack Notch activity. We then analyzed the expression of Senseless and Cut, as readouts of Wg and Notch activity, respectively. Boundary cells lacking Notch activity express Senseless (Figures 3E' and 3F'). Note that in all cases the expression landscape obtained is in excellent agreement with the *in vivo* results (cf. Figures 3C–3F and 3C'–3F').

Finally, when our *in silico* wing primordium lacks Cut activity (either in clones of mutant cells or in a mutant background), boundary cells start responding to the Wg signal, as shown by the expression of Senseless (Figures 4A' and 4B') and ectopic activation of Notch in non-boundary cells is now unable to block the Wg signaling pathway (Figure 4C'). Also, ectopic expression of Cut in non-boundary cells blocks the Wg pathway (Figure 4D'). These *in silico* expression landscapes are also in perfect agreement with the *in vivo* results (cf. Figures 4A–4D and 4A'–4D'; see also Movie S1).

In summary, the results of all the *in silico* experiments are consistent with their *in vivo* counterparts, thus corroborating the regulatory network proposed in Figure 6A.

### *In silico* dynamics of the regulatory network: refinement of Notch activity

**Figure 7:**
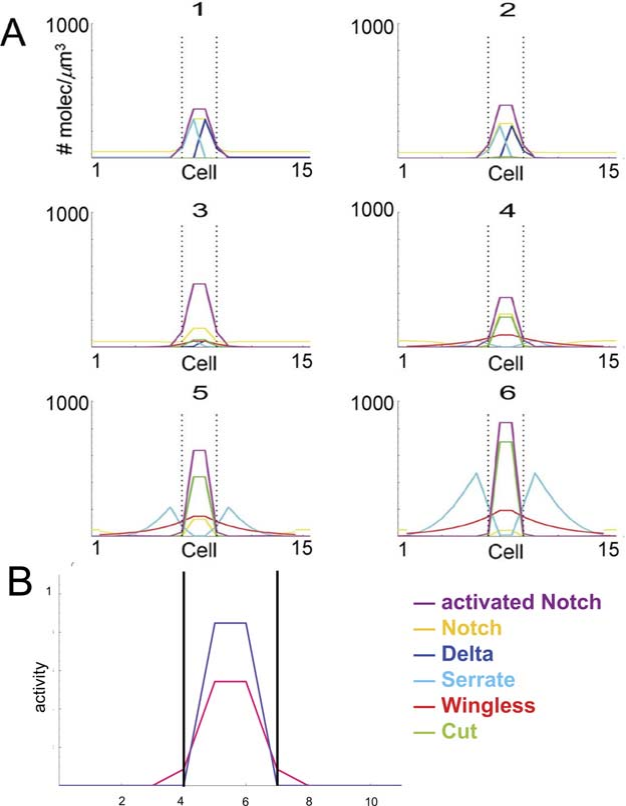
Dynamic process leading to DV boundary establishment and maintenance. (A) Snapshots of the concentration of species versus cell-number along an axis perpendicular to the DV border (dorsal is on the left and ventral on the right). The sequence shows how the expression pattern is generated from the initial condition (1) to the stationary state (6). Frame-to-frame time lapses differ. Dotted lines delimit a four-cell-wide region around the DV boundary and highlight the refinement process (see text). (B) Comparison between the Notch activity domains at different stages (early stages in pink, late stages in purple) that stress the refinement process.

Further insight into the underlying mechanism that leads to the formation of the DV organizer can be gained by a careful analysis of its dynamics. In this regard, note that in a general context, the same stationary pattern can be reached by a number of mechanisms [43], [44]. Thus, proper modeling must reflect not only a stationary final situation but also the dynamics that leads to its establishment. As a novel feature of the study, we perform such a temporal-dependent analysis in our *in silico* experiments. As previously mentioned, a distinctive feature of the process that leads to stable localization of the Notch-dependent organizer at the DV boundary is the refinement of the Notch activation domain to a thin stripe with a final width of two-three cells. Here we analyzed this process with the aid of our *in silico* model, as shown in Figure 7.

Early Notch activity at the boundary first induces expression of the Notch ligands and the Notch receptor. Once Notch activity is large enough, Wg expression is induced. Wg protein diffuses and starts to establish a protein gradient centered on the DV boundary that induces symmetric expression of the Notch ligands Delta and Serrate. The enlargement of the Serrate and Delta expression domains leads to the spreading of the Notch activity domain. Wg counteracts this spreading by repressing the activity of the Notch receptor. Consequently, the activity landscape of Notch shrinks. This spreading-shrinking dynamics is concomitant with a fall and rise in Notch activity levels in boundary cells and is commonly known as refinement. The Notch activation domain is finally confined to cells where Cut is expressed. Snapshots of this evolution are given in Figure 7A. In order to show the dynamics of the expression pattern from the initial condition to the final stationary state more clearly, we provide a movie as Supporting Information (Movie S2; compare with Figure 3B). In addition, Figure 7B highlights the refinement process by combining Notch activity profiles at early and late stages.

### Robustness analysis of the regulatory network

We also validated the proposed regulatory network in terms of its robustness with respect to random, yet biologically plausible, static and dynamic perturbations. We refer the reader to the Protocol S3 of Supporting Information for a detailed description of the robustness analysis technique and its results. Regarding the static robustness analysis (parameters variation), we find that some degree of cooperativity at the level of regulatory interactions is required for the establishment of a robust DV boundary. The *in silico* results presented here correspond to regulatory interactions performed by means of Hill functions with a cooperativity degree equal to 2 (degree equal to 1 corresponds to a Michaelis-Menten dynamics, which is no cooperativity). For this degree of cooperativity, our results show that, on average, the network tolerates up to 91% for single parameter variation according to the assumed Gaussian stochastic distributions. Nonetheless, we noted that even if a Michaelis-Menten dynamics is assumed, the network is also quite robust, allowing 75% for single parameter variation. On the other hand, at the limit of infinite cooperativity, i.e. Boolean regulatory interactions, the tolerance reaches 89%. In spite of the cooperativity exponent, we checked and concluded that there is no differential sensitivity to a particular parameter or set of parameters.

The requirement of cooperativity in the modeling approach for an increased robustness can be understood in terms of an effective regulation between species. The regulatory network that we propose takes into account a reduced set of elements and interactions. That is to say, intermediate “actors”, which nonetheless are known to play a role, have been removed; e.g. Dishevelled, which mediates the negative regulation of the Notch pathway caused by Wg activity. Moreover, in our modeling approach transcription and translation are considered as a single step. When adiabatically eliminated, intermediate actors and processes contribute effectively to the interaction between species in the mathematical modeling via the cooperativity exponent.

Finally, we also analyzed the behavior of the regulatory network when perturbations are dynamically included by means of additive spatio-temporal uncorrelated noise to the species productions (see Protocol 3 in the Supporting Information for details). These noisy contributions randomly vary the species production from cell to cell (independently) during evolution. We note that in this case the network also allows for a robust behavior.

### Concluding Remarks

Here we analyzed the properties of the regulatory network for the establishment and maintenance of the DV organizer in the *Drosophila* wing imaginal disc. We provide evidence that our mathematical model can convert the initial DV asymmetric expression pattern of Notch ligands into the DV symmetric and mutually exclusive domains of active receptor and Notch ligands in boundary and non-boundary cells, respectively. To model the network “circuitry”, and test and verify our proposal, we took advantage of a combination between in vivo and in silico experiments that has allowed us to check the analytical and predictive capacity of our modeling.

The most striking finding of our research is that a novel property is required in the regulatory network for a robust and stable maintenance of the DV organizer: namely boundary cells must be refractory to the Wg signal. This property is conferred by the activity of Notch through its target gene *cut*. The role of Cut in repressing the Wg signaling pathway in boundary cells, and Wg in repressing Notch in non-boundary cells, generates two mutually exclusive domains of Notch and Wg activities, corresponding to boundary and non-boundary cells, respectively. Consequently, Notch ligands and receptors are expressed in two distinct non-overlapping cell populations. This helps to restrict the width of the boundary population to few (two-three) cells and contributes to polarizing ligand-receptor signaling towards the boundary and not against it, i.e. flanking ligands signal Notch *towards* the boundary but not *against* it since down-regulation of the Notch pathway in non-boundary cells inhibits the receptors' activity in those cells. In addition, we have also shed light on several dynamical properties of the network, such as the refinement of Notch activity.

At the time the role of Cut in the repression of Delta and Serrate expression was described [9], Cut and the concomitant restriction of ligand expression to non-boundary cells were postulated to be essential for the stability of the DV boundary [10]. However, the other negative input of Wg into the Notch pathway through the activity of Dishevelled was not taken into account [17]. Our *in silico* results have predicted that a general repression of the Wg pathway is required for stable activity of Notch at the DV boundary. Our in vivo results indicate that this repression takes place at the level or upstream of Armadillo. In order to be refractory to the inhibitory effect of Dishevelled on Notch, this repression should be taking place close to Dishevelled if not further upstream in the Wg signaling cascade.

Finally, we wish to place our conclusions into a broader context. Boundary formation between adjacent rhombomeres in vertebrates relies on the same Wnt/Notch-dependent regulatory network [8]. Therefore, we speculate that boundary cells also need to be refractory to the Wnt signal to generate stable boundaries. To close, we conclude that the robustness and stability of this network, in which the interconnectivity of the elements is crucial and even more important than the value of the parameters used, might explain its use in boundary formation in other multicellular organisms.

## Materials and Methods

### *In vivo* experiments

#### a) *Drosophila* Strains

*cut^6^, cut^C145 ^and UAS-cut* are described in [45]. *UAS-Dl^DN^* is described in [36]. *UAS-Arm^S10^, UAS-dFz2, UAS-dsh, ptc-Gal4, dpp-Gal4, UAS-Wg, UAS-N^intra^* and *N^co^* are described in Flybase. The following *Drosophila* genotypes were used to generate loss-of-function clones: *N^co^ FRT18A/arm-lacZ FRT18A; hs-FLP/+* and *cut^C145^ FRT18A/arm-lacZ FRT18A; hs-FLP/+.* Larvae were heat shocked for 1 h at 37°C.

#### b) Antibodies

Monoclonal antibodies against the following proteins are described in the Developmental Studies Hybridoma Bank: Notch (intracellular domain), Cut, Armadillo, Delta and Wingless. Guinea-pig anti-Senseless is described in [37] and was kindly provided by H. Bellen. Other antibodies are commercially available.

### *In silico* experiments

#### a) Numerical scheme

We performed our *in silico* experiments in a two-dimensional hexagonal lattice by means of an explicit first-order forward-time-centered-space scheme with time step 10^−2^s and size 50×30. Each lattice node represents a cell and therefore the *in silico* imaginal disc comprises 1500 cells. For this lattice size, we checked that the imposed boundary conditions did not introduce any artifact in our simulations.

#### b) Modeling equations

By taking into account the “circuitry” regulatory interactions shown in Figure 6A, and the modeling scheme indicated in Protocol S1 (Supporting Information), the differential equations that represent the network read,
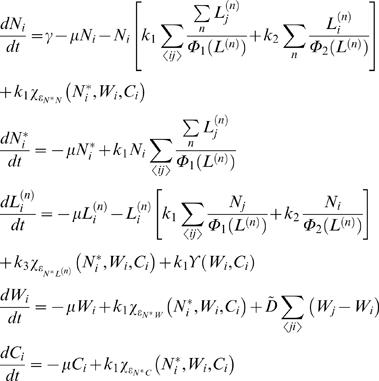
where N, N^*^, *L^(n)^*, *W*, and *C* represent Notch receptor, activated Notch, Ligands, Wingless, and Cut respectively. Species subscript *i* stands for cell labeling. For the sake of concision, the following functions have been defined,
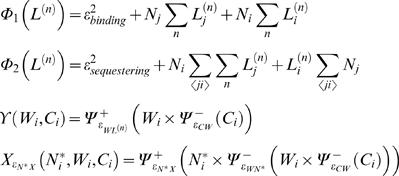
Functions *Ψ_ε_* ^±^(*x*) indicates positive/negative Hill regulatory functions (see Protocol S1 for details), whereas 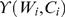 and 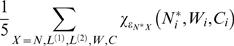 account for the activities of Wingless and Notch pathways respectively (0 to 1 scale). The former has been used as the Wingless activity reporter Senseless. As for the latter, we found that is qualitatively similar to consider the activated Notch species as a reporter of Notch activity (data not shown). Note that there are 6 differential equations since the superscript *n* takes the values 1 and 2 depending of the ligand. Wingless diffusion was included by means of a discrete version of a Laplacian operator. Note also that the first term that appears on the right hand side of the equation for Notch dynamics, *γ*, accounts for cell-autonomous off-network expression.

## Supporting Information

Figure S1 (A) Mature wild-type (A) wing disc showing Delta protein expression (in red) in non-boundary cells. (B) Mature wing disc expressing Wingless (Wg) under *dpp* ^Gal4^ control and labeled to visualize Delta protein expression (in red). (C) Mature *cut* ^6^ mutant wing disc showing Delta protein expression (in red) in boundary cells. (D) Clone of cells lacking *cut* activity and marked by the absence of GFP (green). Delta protein (in red) starts to be expressed in boundary cells.
(6.80 MB TIF)

Figure S2 (A) “Toy” regulatory network: Gene-protein B is positively regulated by gene-protein A. On the other hand, this interaction is negatively regulated by gene-protein C. Hill functions, with a given degree of cooperativity, *β*, are assumed to effectively model gene-protein regulation. (B) Hill functions for distinct values of cooperativity. As *β* increases, regulation becomes stiffer and the Hill functions tend towards step functions. Both positive (B top) and negative (B bottom) regulatory functions depend on the concentration of inducer/repressor species x.
(0.39 MB TIF)

Figure S3 (A) Evolution of Wg (red) expression levels, Notch activated (black), and Cut (green) in boundary cells as a function of time for the regulatory scheme shown in Figure 6A. The boundary is established and maintained. (C) *In silico* evolution of the patterns of distinct species. This pattern is in agreement with in *vivo* results (compare with Figure 3B). The DV boundary is marked by a black arrowhead.
(0.49 MB TIF)

Figure S4 Histograms (count, i.e. not normalized) of the parameter values used in the ∼1.5 10.4^4^ *in silico* experiments used in the robustness analysis. Units depend on the quantity depicted (see text). The initial condition was also subjected to variation (data not shown).
(0.82 MB TIF)

Movie S1 The movie shows the evolution of expression levels of species in the absence of Cut (no refractoriness to the Wg signal) (*in silico*).
(1.89 MB MOV)

Movie S2 The movie shows the evolution of expression levels of species in a wild-type background (*in silico*).
(1.88 MB MOV)

Protocol S1 (0.07 MB DOC)

Protocol S2 (0.08 MB DOC)

Protocol S3 (0.03 MB DOC)
