# Supplementary figures and images for: Robustness and Stability of the Gene Regulatory Network Involved in DV Boundary Formation in the Drosophila Wing
Source: PLoS One. 2007 Jul 11;2(7):e602. doi: 10.1371/journal.pone.0000602 (PMC1904254; doi:10.1371/journal.pone.0000602)

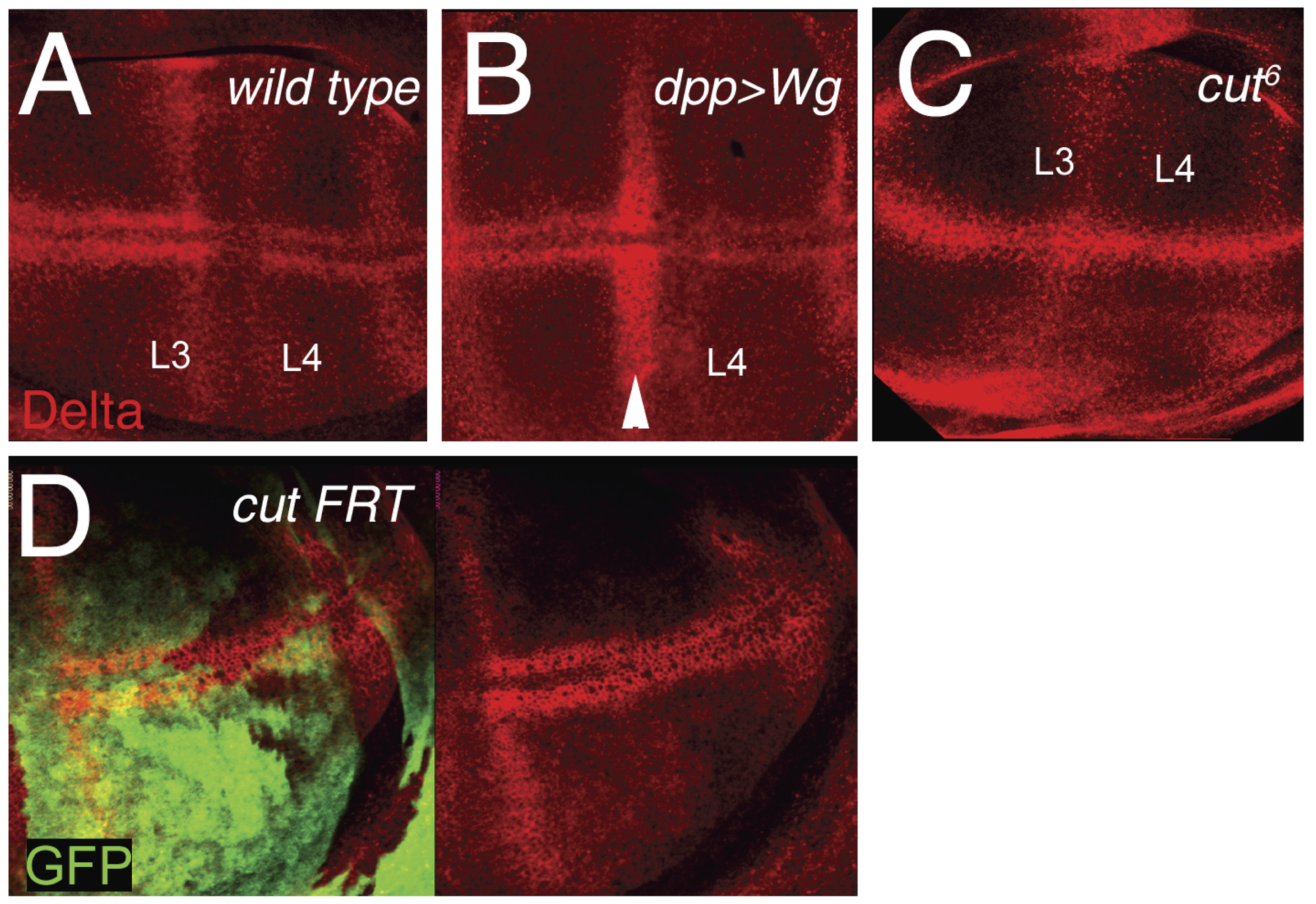

Supplement: Figure S1 — (A) Mature wild-type (A) wing disc showing Delta protein expression (in red) in non-boundary cells. (B) Mature wing disc expressing Wingless (Wg) under dpp Gal4 control and labeled to visualize Delta protein expression (in red). (C) Mature cut 6 mutant wing disc showing Delta protein expression (in red) in boundary cells. (D) Clone of cells lacking cut activity and marked by the absence of GFP (green). Delta protein (in red) starts to be expressed in boundary cells. (6.80 MB TIF) [file pone.0000602.s001.tif]

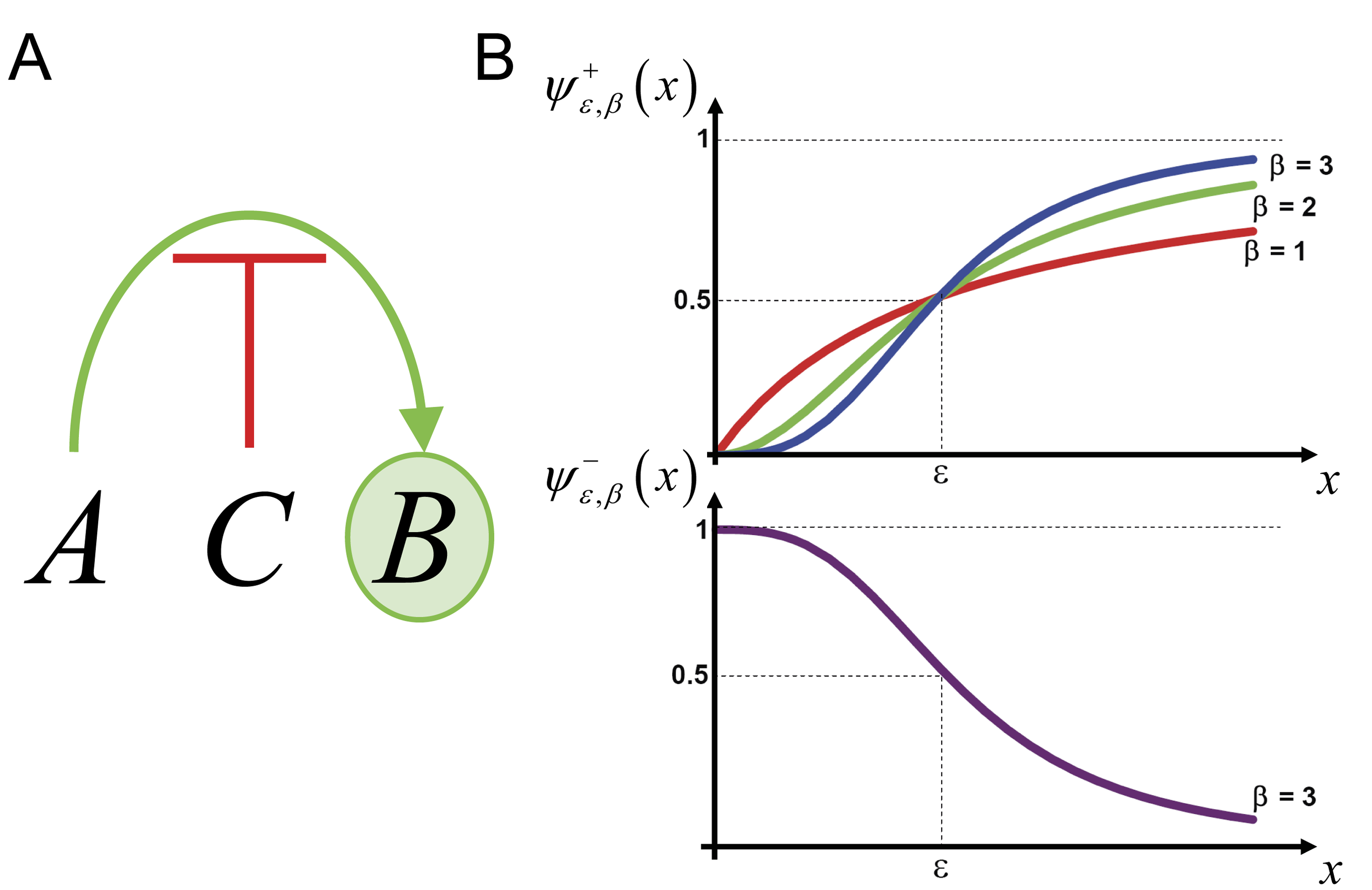

Supplement: Figure S2 — (A) “Toy” regulatory network: Gene-protein B is positively regulated by gene-protein A. On the other hand, this interaction is negatively regulated by gene-protein C. Hill functions, with a given degree of cooperativity, β, are assumed to effectively model gene-protein regulation. (B) Hill functions for distinct values of cooperativity. As β increases, regulation becomes stiffer and the Hill functions tend towards step functions. Both positive (B top) and negative (B bottom) regulatory functions depend on the concentration of inducer/repressor species x. (0.39 MB TIF) [file pone.0000602.s002.tif]

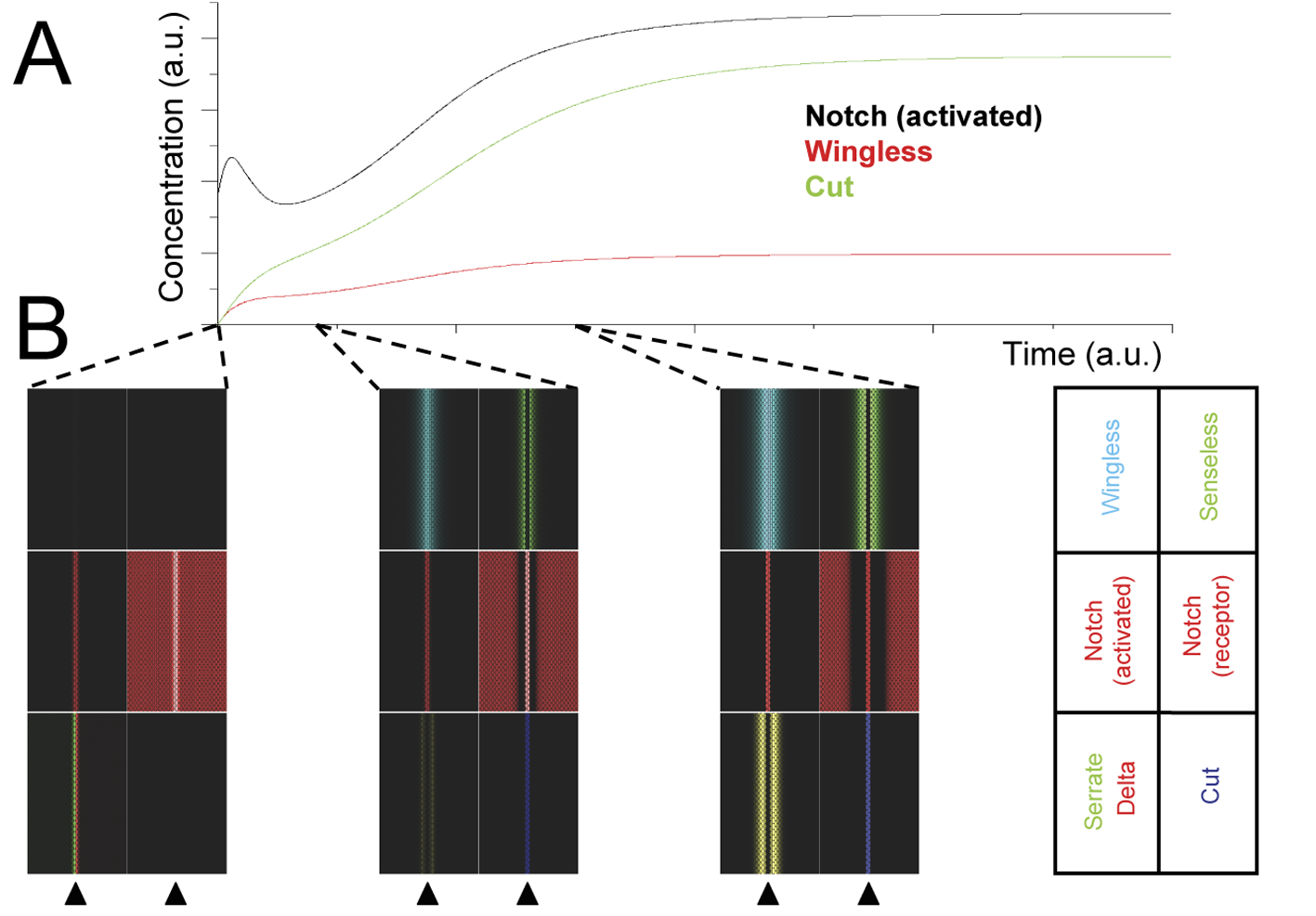

Supplement: Figure S3 — (A) Evolution of Wg (red) expression levels, Notch activated (black), and Cut (green) in boundary cells as a function of time for the regulatory scheme shown in Figure 6A. The boundary is established and maintained. (C) In silico evolution of the patterns of distinct species. This pattern is in agreement with in vivo results (compare with Figure 3B). The DV boundary is marked by a black arrowhead. (0.49 MB TIF) [file pone.0000602.s003.tif]

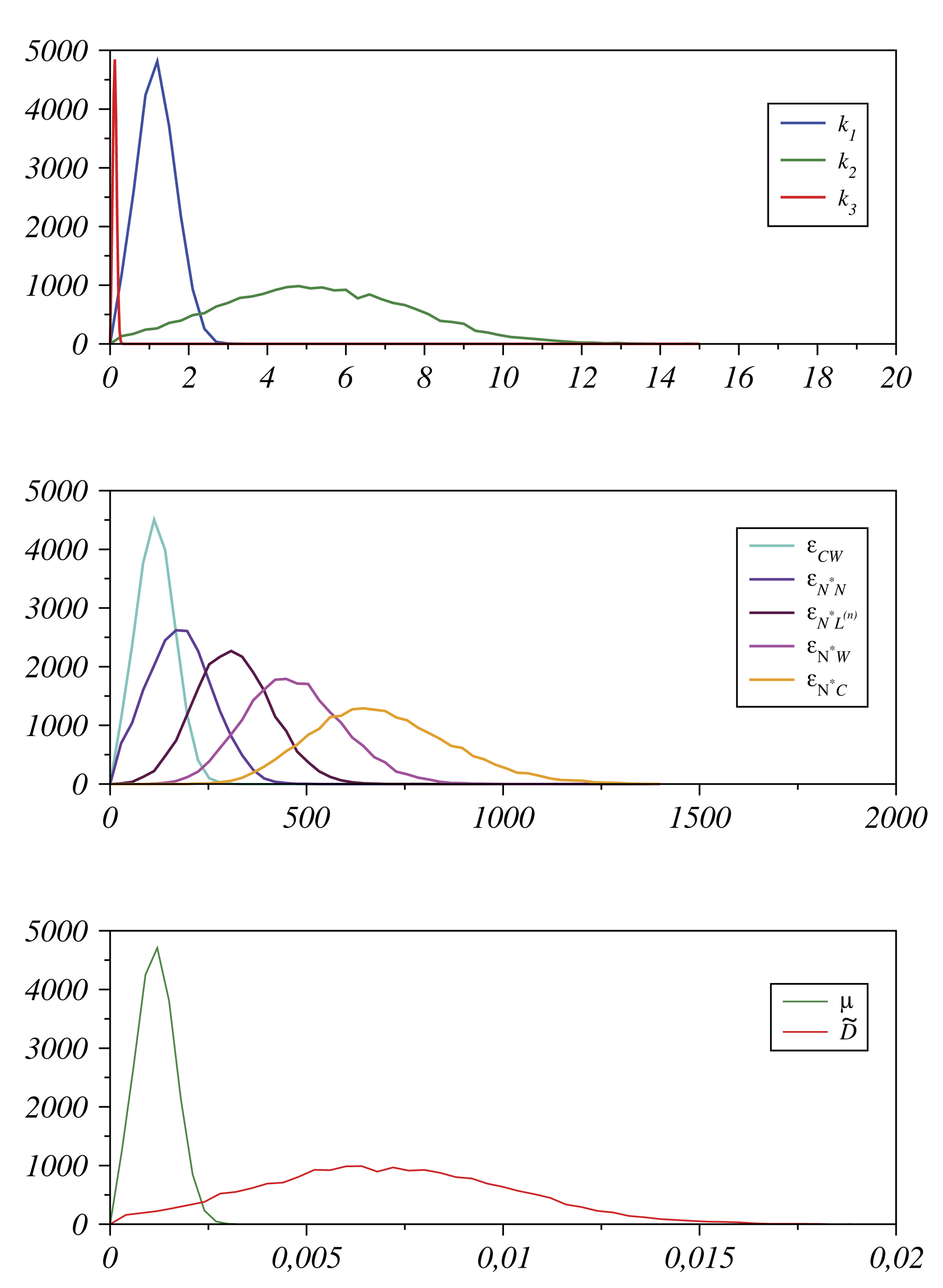

Supplement: Figure S4 — Histograms (count, i.e. not normalized) of the parameter values used in the ∼1.5 10.44 in silico experiments used in the robustness analysis. Units depend on the quantity depicted (see text). The initial condition was also subjected to variation (data not shown). (0.82 MB TIF) [file pone.0000602.s004.tif]
