# Supplementary material for: Robustness and Stability of the Gene Regulatory Network Involved in DV Boundary Formation in the Drosophila Wing
Source: PLoS One. 2007 Jul 11;2(7):e602. doi: 10.1371/journal.pone.0000602 (PMC1904254; doi:10.1371/journal.pone.0000602)
Supplement: Protocol S1 — (0.07 MB DOC) [file pone.0000602.s007.doc]

**Protocol S1: Modeling approach and ligand-receptor dynamics**

We implemented a modeling scheme where the species of interest are the concentration of proteins or protein-related products, e.g., a biomolecule resulting from protein cleavage. For the sake of simplicity, our modeling approach reduced each transcriptional-translational dynamics of a gene regulatory network into a single effective process where Hill-like functions, with a given degree of cooperativity, are assumed as regulatory functions. We disregarded time delays between transcription and translation. These time lags have been proved crucial in other developmental processes [1]. However, as we have verified, this is not the case here. The resulting differential equations mimic the temporal behavior for the concentration of proteins, and/or other protein-related products in a cell as a consequence of gene interactions.

Figure S2 illustrates the regulatory interactions between three genes and shows schematic representations of positive and negative regulatory Hill functions. Thus, for this “toy” network, the regulation of gene-protein *B* caused by interactions with genes-proteins *A* and *C* reads,

$\psi_{\varepsilon_{AB}}^{+}\left(A\times\psi_{\varepsilon_{BC}}^{-}\left(C\right)\right)$ (1)

Where the positive, $\psi_{\varepsilon}^{+}\left(x\right)$, and negative, $\psi_{\varepsilon}^{-}\left(x\right)$, regulatory functions read, respectively,

$\begin{matrix} \psi_{\varepsilon}^{+}\left(x\right)=\frac{x^{\beta}}{\varepsilon^{\beta}+x^{\beta}}, \\ \psi_{\varepsilon}^{-}\left(x\right)=1-\psi_{\varepsilon}^{+}\left(x\right). \end{matrix}$ (2)

That is, Hill functions with a given degree of cooperativity, *β,* are assumed to effectively model interactions between species. The larger *β,* the stronger the cooperativity and the steeper the transition between states 0 and 1. The parameter *ε* measures the concentration threshold of a given protein, $x$, for which the regulatory interaction, either positive or negative, reaches *50%* intensity (see Figure S2).

We also considered degradation for species by means of exponential decays for all of them. Note that robustness in morphogen gradient profiles can be increased by means of self-enhanced degradation terms, thus leading to power-law decays [2]. For the sake of simplicity, in our modeling approach we disregarded these contributions.

Therefore, the modeling differential equation for species *B* becomes,

$\frac{dB}{dt}=k_{B}\times\psi_{\varepsilon_{AB}}^{+}\left(A\times\psi_{\varepsilon_{BC}}^{-}\left(C\right)\right)-\mu_{B}\times B$ (3)

where $k_{B}$ and $\mu_{B}$ stand for the expression and degradation rate constants, respectively. As explained below, we also took into account diffusion and cell-autonomous transcription-translation dynamics for some species. The former is a crucial element of morphogen kinetics whereas the latter is required to reproduce basal levels of expression in a cell-autonomous way.

Inter- and intra-cellular interactions caused by receptor-ligand dynamics were modeled as follows. The activation of the receptor in a given cell takes place exclusively when it binds to a ligand that belongs to a (nearest) neighboring cell. Receptor-ligand binding events within the same cell certainly happen. However, in these cases no activation of the receptor is produced and both receptor and ligand are “sequestered” and become useless for further signaling purposes. For simplicity, we did not consider unbinding dynamics. If successful, the activation of the receptor produces proteolytic cleavage of its intracellular part, which translocates to the nucleus where it induces the expression of downstream genes. Let us denote$x_{i}$, $x_{i}^{*}$, and $y_{i}$ the receptor, its intracellular active part, and the ligand concentrations at cells *i* and *j,* respectively. We momentarily disregarded upstream and downstream transcription-translation processes and degradation. Accordingly, the receptor-ligand regulatory dynamics reads,

$\begin{matrix} \frac{dx_{i}}{dt}=-x_{i}\cdot\left[k_{binding}\cdot\sum_{\left\langle ij\right\rangle} \frac{y_{j}}{\varepsilon_{binding}^{2}+x_{j}\cdot y_{j}+x_{i}\cdot y_{i}}+k_{sequestering}\cdot\frac{y_{i}}{\varepsilon_{sequestering}^{2}+x_{i}\cdot\sum_{\left\langle ij\right\rangle} y_{j}+y_{i}\cdot\sum_{\left\langle ij\right\rangle} x_{j}}\right], \\ \frac{dy_{i}}{dt}=-y_{i}\cdot\left[k_{binding}\cdot\sum_{\left\langle ij\right\rangle} \frac{x_{j}}{\varepsilon_{binding}^{2}+x_{j}\cdot y_{j}+x_{i}\cdot y_{i}}+k_{sequestering}\cdot\frac{x_{i}}{\varepsilon_{sequestering}^{2}+x_{i}\cdot\sum_{\left\langle ij\right\rangle} y_{j}+y_{i}\cdot\sum_{\left\langle ij\right\rangle} x_{j}}\right], \\ \frac{dx_{i}^{*}}{dt}=+x_{i}\cdot\left[k_{binding}\cdot\sum_{\left\langle ij\right\rangle} \frac{y_{j}}{\varepsilon_{binding}^{2}+x_{j}\cdot y_{j}+x_{i}\cdot y_{i}}\right]. \end{matrix}$(4)

where $\left\langle ij\right\rangle$ indicates that the sums in Eq.(4) run over all cells *j* that are the nearest neighbors of cell *i*. Note also that receptor-ligand dynamics is prescribed in terms of Hill-like regulatory functions. Note that activation events are conveniently weighted in the regulatory functions depending on the number of receptor-ligand couples that can be created within the same cell since they will reduce the probability of successful cell-cell bindings. Likewise, the sequestering events are weighted by taking into account the number of receptor-ligand couples that are formed between cells since they decrease the probability of a sequestering event. Altogether, this dynamics may lead to either positive or negative regulation of $x_{i}^{*}$ depending on the local concentrations of $x_{i}$ and$y_{i}$, and on the values of the parameters *k* and *ε*. As shown below, a generalization of the modeling equations when the receptor can be signaled by several ligands is straightforward.

By taking into account the aforementioned considerations and the “circuitry” shown in Figure 6, we obtain the modeling equations shown in Material and Methods Section (*in silico* experiments: Modeling equations). Modeling equations for other regulatory schemes, e.g. Figure 2A, can be obtained in a similar fashion. Finally, we note that cell proliferation and motility have been disregarded. The former plays a key role in subsequent developmental stages when the border has been already established but can be omitted within the temporal window of our interest. As for the latter, it can be ignored altogether within this context.
