# Supplementary material for: Robustness and Stability of the Gene Regulatory Network Involved in DV Boundary Formation in the Drosophila Wing
Source: PLoS One. 2007 Jul 11;2(7):e602. doi: 10.1371/journal.pone.0000602 (PMC1904254; doi:10.1371/journal.pone.0000602)
Supplement: Protocol S2 — (0.08 MB DOC) [file pone.0000602.s008.doc]

**Protocol S2: Parameter estimation**

We kept the set of parameters as small as possible. However, we maintained a realistic approach and therefore this set should be large enough to take into account well-known biological events. For example, ligand expression rates caused by Notch activity are known to be smaller than those caused by Wingless. Similarly, at large concentration regimes, sequestering effects play a relevant role in receptor-ligand dynamics when compared to binding. Thus, as shown in our modeling equations, whereas the degradation rate constant,$\mu$, was kept the same for all species, three regulation rate constants were used:$k_{1}$(binding and the rest of gene/protein regulatory constants apart from ligand expression caused by Notch activity), $k_{2}$(sequestering), and $k_{3}$(ligand expression caused by Notch activity).

To the best of our knowledge, most of the parameter values have not been measured previously. However, very recent experiments on the kinetics of morphogen gradient formation have quantitatively characterized some [3]. As for the Wg diffusion rate, a value of *~*0.1*μm^2^/s* has been reported. This value is significantly smaller than previously reported measurements:$\sim1.4\mu m^{2}/s$ [4]. The value of the diffusion rate used in our *in silico* experiments is closer to the latter (see below). Furthermore, the degradation rate for Wg has been estimated to be *~*10^-3^*s^-1^*. In other systems the protein degradation rates ranges from $10^{-6}s^{-1}$ to $10^{-2}s^{-1}$. Herein we stick to $10^{-3}s^{-1}$. As for the effective transcription-translation rates, we estimated them as follows: if a given species is subjected to regulation and degradation, e.g. Eq.(3), then the maximum value of its concentration in the steady-state will be given by (note that the regulatory functions are dimensionless),

$\left.A_{s}\right|_{\max}=\frac{k_{A_{2}}}{\mu_{A_{2}}}$. (5)

Obviously, the minimum value is zero. Therefore, if the degradation rates and the steady concentration of protein are known amounts, then the effective transcription-translation rates can be estimated. The number of proteins in a cell commonly ranges from $10^{4}$ to $10^{7}$. By taking into account that the typical diameter of a cell is $10\mu m$, we found that $k\in\left(\sim10^{-1},\sim10\right)proteins/\left(\mu m^{3}\cdot s\right)$.

According to the robustness analysis, some degree of cooperativity, *β*, is mandatory: we set *β*=2. The thresholds for regulation, *ε*’s, and the fine-tuning and estimation of the parameters were obtained by means of *in silico* experiments. These experiments allowed us to knockout or over-express a gene, or a set of genes, for a particular group of cells. Thus, the behavior observed within the clones and in neighboring cells for *in silico* experiments and the comparison with their *in vivo* counterparts allowed us to check whether the gene interactions were appropriately defined and weighted (see below). By testing several clones, we converged a set of parameter values that reproduces the wild-type behavior and the clonal analysis therefore estimating their values. Tables 1 and 2 summarize the values used in our modeling approach for *k*’s and *ε*’s parameters.

The values of parameters *γ* (Notch basal transcription-translation) and $\tilde{D}$ (diffusion parameter) were set as follows. For the former, *γ* was set to $5\cdot10^{-2}proteins/(\mu m^{3}\cdot s)$ thus ensuring a minimum number of receptors at each cell, $\sim50proteins/\mu m^{3}$. For the diffusion, we first noted that discretization of the Laplacian operator for a two-dimensional hexagonal lattice leads to,

$D\nabla^{2}\psi\to\frac{2}{3}\frac{D}{l^{2}}\sum_{\left\langle ij\right\rangle} \left(\psi_{j}-\psi_{i}\right)$, (6)

where $l$ is the lattice spacing, i.e., the typical cell size, 10*μ*m, and the sum runs over the nearest-neighbors. Notice that in our modeling equations we defined $\tilde{D}=2D/\left(3l^{2}\right)$. The value of the latter was $\tilde{D}\approx7\cdot10^{-3}s^{-1}$, which corresponds to a “true” diffusion coefficient of $D=1\mu m^{2}/s$.

Finally, for the initial condition, we initially divided our *in silico* imaginal disc into two domains, which corresponded to D and V compartments. This division is characterized by the initial asymmetric expression pattern of the ligands. The value of the initial concentrations of ligands in the disc pouch is very small in most cells, $10proteins/\mu m^{3}$. However, in boundary cells, a larger concentration of ligands is expected as a result of the positive feedback induced by Apterous onset at previous developmental stages. Moreover, in boundary cells an initial concentration of Notch and activated Notch is also expected. We set their values to $300proteins/\left(\mu m^{3}\right)$and $360proteins/\left(\mu m^{3}\right)$ respectively. We also included one fourth of these amounts as initial concentrations of Notch and activated Notch in the neighboring cells nearest to the boundary. Wg and Cut concentrations were initially set to zero.

We performed ectopic expression and loss-of-function experiments in clones of cells, and also mutant genotype experiments. Ectopic expression was achieved by adding an extra off-network positive regulatory term to the species to clone cells, i.e. a *γ* term like that in the modeling equations for the receptor. We set its value to$3proteins/\left(\mu m^{3}\cdot s\right)$. As for lack-of-function or mutant genotypes, we simply set the production of the species under consideration to zero.

**Table 1** . ***In silico* experiments: *ε*’s values used for numerical simulations.**

| $subscript$ | $N^{*}N$ | $N^{*}L^{\left(n\right)}$ | $N^{*}W$ | $N^{*}C$ | *Bind.* | *Seq.* | $WN^{*}$ | $CW$ |
| --- | --- | --- | --- | --- | --- | --- | --- | --- |
| $\varepsilon\left(\frac{proteins}{\mu m^{3}}\right)$ | *200* | *300* | *400* | *500* | (*50000*)*^1/2^* | (*1000*)*^1/2^* | *100* | *100* |

**Table 2. *In silico* experiments: *k***’s values used for numerical simulations.

| $subscript$ | $1$ | $2$ | $3$ |
| --- | --- | --- | --- |
| $k\left(\frac{proteins}{\mu m^{3}\cdot s}\right)$ | *1* | *5* | *0.1* |
