# Supplementary material for: Robustness and Stability of the Gene Regulatory Network Involved in DV Boundary Formation in the Drosophila Wing
Source: PLoS One. 2007 Jul 11;2(7):e602. doi: 10.1371/journal.pone.0000602 (PMC1904254; doi:10.1371/journal.pone.0000602)
Supplement: Protocol S3 — (0.03 MB DOC) [file pone.0000602.s009.doc]

**Protocol S3: Robustness analysis**

**Static analysis: parameter variation.** We implemented a Gaussian distributed random variation of each parameter around their values, as reported above and shown in Table 1 of Protocol S2. The dispersion typically allows a *50%* variation. We stress that each parameter was treated separately but consistently, e.g., terms where $k_{1}$ appears are subjected to an independent random variation around its mean value. Note that since the Gaussian distribution is unbound, negative values can be obtained: we obviously disregarded these in our analysis. Moreover, we checked that each parameter set ensured biological realism in the following regard. The Notch activity threshold sequence to induce regulation of downstream genes is experimentally well-known and a component of the DV boundary formation mechanism. Therefore, a valid parameter set must fulfill the following condition,

$\varepsilon_{N^{*}C}>\varepsilon_{N^{*}W}>\varepsilon_{N^{*}L^{\left(n\right)}}>\varepsilon_{N^{*}N}$ (7)

This procedure implies that the distributions generated for these amounts are not kept independent. By maintaining the cooperativity parameter *β* unaltered, we generated $\sim1.5\cdot10^{4}$ valid parameter sets. We then performed *in silico* experiments for each set and checked whether the main features of the formation of the DV boundary were correctly reproduced (size, expression pattern, polarized signaling, etcetera). Afterwards, we repeated the *in silico* tests for the same sets of parameters but changing *β*. This procedure allowed us to test the importance of cooperativity in the proposed regulatory network. Figure S4 shows the parameter distributions used in the robustness analysis. The values used for *β*, apart from *β=2*, either disregarded cooperativity, *β=1*, or overestimated (with respect to our original guess) its value: *β=* (Boolean interactions). For the results obtained, we evaluated the ratio, $r_{\beta}$, between successful outcomes and the total number of experiments.

As $r_{\beta}\to0/1$, the system becomes less/more robust. We also computed $\left(r_{\beta}\right)^{1/n}$, where $n$ is the size of our parameter set, i.e., the number of parameters we allowed to vary independently: 23 in our case. Note that the initial condition was also taken into account in our robustness analysis. This quantity, $\left(r_{\beta}\right)^{1/n}$, provides information on the degree of robustness of each parameter: if all parameters but one are kept unaltered, it measures the degree of robustness for single parameter variation. We obtained,

(*r_β=1_*) ^1/23^0.75; (*r_β=2_*) ^1/23^0.91; (*r_β=_*)^1/23^0.89 (8)

We compared the distributions of parameters for successful and unsuccessful outcomes with respect the stochastic distributions used in the robustness analysis and found that, in spite of the cooperativity exponent, there is no differential sensitivity to a particular parameter, or set of parameters, in either case (data not shown).

**Dynamic analysis: production variation.** We also perform a robustness analysis with respect to the production rates. To this end, we included additive noise contributions to our modeling equations. We consider Gaussian white noises in space and time (zero mean, uncorrelated in space and time). Since white noise is unbounded, we checked that these contributions do not include artifacts in our simulations as for example a negative value of the concentrations. Under these conditions, the system behaves robustly with respect to noisy contributions with an intensity up to order one. This intensity implies an average production variation up to *~*±5 *proteins*/(*μm^3^s)*at any given cell and time i.e. two orders of magnitude larger (in absolute value) than the basal production rate of receptors.

**References**

1. Raya A, Kawakami Y, Rodriguez-Esteban C, Ibanes M, Rasskin-Gutman D, et al. (2004) Notch activity acts as a sensor for extracellular calcium during vertebrate left-right determination. Nature 427: 121-128.

2. Eldar A, Rosin D, Shilo BZ, Barkai N (2003) Self-enhanced ligand degradation underlies robustness of morphogen gradients. Dev Cell 5: 635-646.

3. Kicheva A, Pantazis P, Bollenbach T, Kalaidzidis Y, Bittig T, et al. (2007) Kinetics of morphogen gradient formation. Science 315: 521-525.

4. Strigini M, Cohen SM (2000) Wingless gradient formation in the Drosophila wing. Curr Biol 10: 293-300.
